# Political party affiliation, social identity cues, and attitudes about protective mask-wearing during the COVID-19 pandemic in Germany

**DOI:** 10.1371/journal.pone.0302399

**Published:** 2024-06-06

**Authors:** Kathleen D. Magnus, Niklas Dammann, Elâ Ziegler, Daniel Lüdecke, Demet Dingoyan

**Affiliations:** Institute of Medical Sociology, University Medical Center Hamburg-Eppendorf, Hamburg, Germany; University of Haifa, ISRAEL

## Abstract

This cross-sectional study aimed to determine 1) whether German citizens’ adherence to health professionals’ recommendations and mandates regarding protective masks during the COVID-19 pandemic varied according to their political party affiliations, and 2) how behavioral cues provided by members of shared social groups, such as family and friends, influenced individual mask-wearing behavior. A quota-based sample of German voters (n = 330) consisting of 55 citizens whose voting intentions aligned with each of the country’s six main political parties responded to an online questionnaire consisting of multiple-choice and open-ended questions. Univariate descriptive statistical analyses of quantitative data were conducted, and multiple regressions were performed to determine log odds and significant variations among group-based responses. A pragmatic inductive coding process was used to conduct a thematic analysis of qualitative data. Results indicated that those participants who expressed an intention to vote for the populist radical right party were the least likely to follow health experts’ recommendations and the most likely to express anger and dissatisfaction over mask mandates. Prospective Left Party voters were the most likely to adhere to the advice of their doctors, while those associated with the Green Party were the most likely to adhere to the advice of public health experts. Most survey participants reported aligning their mask-wearing behavior with that of family and friends, with prospective CDU/CSU voters particularly likely to consider the mask-wearing behavior of family members. The results indicate that public health officials should consider how group-related factors influence public health compliance in order to encourage protective mask-wearing in the future.

## Introduction

Public health measures designed to mitigate the spread of the Covid-19 virus were controversial in Germany, as in many Western countries [1]. One reason for this may have been the unequal economic, social, and emotional burdens that these measures entailed for different sectors of society. Lockdowns, for instance, had serious economic consequences for some citizens (i.e., loss of work, reduced pay), while they granted unprecedented freedoms to others (i.e., flexible work hours through home office). The social and mental health effects of lockdowns also varied greatly, as those who lived alone were forced to experience the most isolation, and certain demographic groups, such as young adults and women, suffered more negative mental health consequences than others [2]. By contrast, mask mandates applied equally to everyone who entered the public sphere, and they offered the opportunity to overcome these lockdown-related burdens. Nonetheless, compliance with mask recommendations and mandates was not optimal [3,4]. Given the high costs of lockdowns, the extended time period needed to develop and distribute vaccinations, and the demonstrated effectiveness of masks in stemming the virus [5,6], protective mask-wearing is likely to play an important role in the mitigation of contagion if there is an outbreak of another airborne virus in the future. For this reason, research identifying factors linked to (non) compliance with mask-wearing recommendations and mandates continues to be of vital importance.

However, German-based studies investigating citizen compliance with COVID-19 mitigating measures have seldom focused on protective mask-wearing, and to our knowledge, there has yet to be an extensive study examining how mask-related health behavior may have been associated with political party affiliation in Germany. Although compliance with mask-wearing was an intensely discussed and highly politicized issue in many Western countries [7], German-based studies that considered political party affiliations in relation to COVID-19-related health behavior more commonly focused on issues related to vaccinations and lockdowns (See, for example [8,9]).

Moreover, those who have discussed the influence of political affiliation in Germany have tended to focus on individuals affiliated with Germany’s far right party, the “Alternative for Germany” (Alternative für Deutschland, “AfD”) or on other, vaguely defined “populists” [10]. Some of these studies documented a high rate of dissatisfaction among AfD voters with regard to the government’s general COVID-19 response [11], as well as a considerable hesitancy to receive COVID-19 vaccinations [12]. Such positions align with the generally anti-establishment and anti-science positions central to the agenda of far-right populist parties [13], and they are in accordance with the positions of several AfD leaders, who, at least during some periods of the pandemic, criticized science-based public health measures, spread conspiracy theories about them, and questioned the government’s authority to enforce measures that limited citizens’ freedoms [14–16].

The extent to which German citizens followed the cues of political elites during the pandemic remains unclear, however [17], and several studies have cast doubt on the idea that this resistance to COVID-19 mitigating measures was primarily a right populist phenomenon [18]. One study found that “ideological extremism on both ends of the spectrum explains skepticism of vaccination” [19]. Another study, which measured the mobilization potential for the anti-corona protests, concluded that most potential protestors came from the political center [20]. Although neither of these studies directly contradicts the conclusion that AfD party affiliates were the most resistant to COVID-19 mitigating measures, they do suggest the need for further clarity on the impact that political party affiliation may have on individuals’ health behavior–especially with regard to their compliance with protective mask-wearing. Our study addressed this need by analyzing survey results from ordinary citizens across the political spectrum.

We have also taken a somewhat unique approach. Studies that have sought to identify factors contributing to compliance with COVID-19 mitigating measures have typically understood compliance in terms of individual character traits, emotions, or values rather than the result of group-based processes. One cross-sectional online survey in China, Germany, and the United States, for instance, considered the effect of various emotions, demonstrating that panic, anxiety, and sadness are associated with greater compliance, while anger and loneliness are associated with less compliance [21]. Other studies have determined individuals’ perceived risk [22], psychological dispositions [23], ideological commitments [24] and basic value orientations [25] to be factors that influenced COVID-19 related health compliance.

In contrast, relatively few studies have considered the role that group processes played with respect to individual COVID-19-related behavior, although there have been some significant contributions in this area. Some research has shown that the pandemic produced conditions conducive to the illusions, ill judgement, and conformity pressures typical of “groupthink” [26]. Other studies emphasized the role of social norms, including one based in Japan, which showed that the perception of mask-wearing as a social norm influenced mask-wearing more positively than the perceived threat of the disease [27].

Our study drew on social psychology to further identify group-based factors. Specifically, we applied the social identity approach (SIA), a set of social psychological theories based on the core insight that the behavior of individuals is significantly influenced by their group identifications. SIA has been established as a useful framework for examining health related questions [28], and an important body of literature stemming from this area has arisen in response to the COVID-19 pandemic [29]. So far, however, few SIA theorists have addressed issues related to compliance with COVID-19 mitigating measures, though there have been some important contributions to this topic outside of Germany [30,31].

Our aims were twofold. First, we sought to determine whether German citizens’ adherence to the advice of health professionals regarding masks during the COVID-19 pandemic varied according to their political party affiliations. Since studies from other countries have established a strong association between mask-wearing compliance and political affiliation [32,33], we hypothesized that a similar correlation could be established among German citizens. Due to the fact that only a small percentage (1.66%) of Germans officially belong to any political party [34], we used hypothetical voting intentions as the marker of group identification, postulating that German voters identify strongly enough with the parties they support to influence their behavior outside the voting booths. Secondly, we aimed to determine whether and to what extent behavioral cues provided by members of shared social groups, such as family and friends, influenced individual mask-wearing behavior. This allowed us to determine whether supporters of each political party followed cues from certain groups more than others.

Our study is unique in its emphasis on the mask-related experiences, views, and behaviors of ordinary German citizens whose voting intentions cut across the political spectrum, as well as in its group-based social identity approach. Although we did not find the extreme political polarization around protective mask-wearing that existed in some countries [35,36], our results indicated some significant variances along party lines.

## Materials and methods

### Questionnaire design

We designed eight multiple-choice questions asking participants to identify the extent to which authorities and social groups influenced and/or corresponded with their mask-wearing behavior. The first two questions asked participants if their behavior corresponded with health authorities, namely with 1) the advice of their doctors and 2) the advice of public health institutions such as the Robert Koch Institute (RKI). Participants could choose one of five responses, indicating the extent to which they considered these authorities and groups: “not so much,” “sometimes,” “always/almost always,” or indicating that they didn’t know or didn’t see the question as relevant. Here it is important to note that while positive responses can be interpreted to signify compliance, an indifferent or negative response cannot necessarily be construed as non-compliance; these merely indicate that the health authorities’ advice was not a factor in making decisions about health behavior–an indication, which is nonetheless telling.

The remaining six questions asked survey participants to consider whether their decision to wear (or not to wear) a mask aligned with the behavior of various social and political groups to which they belonged. Here the point was not to gather evidence about compliance or non-compliance with mask recommendations and regulations, but rather to determine the extent to which individuals from each party affiliation took behavioral cues from 3) friends and acquaintances; 4) family members; 5) colleagues at work, school, or the university; 6) members of shared community groups, such as sport clubs and churches; 7) members of their own political party; and/or 8) others who share similar political views. Participants could choose one of five responses: “not so much,” “sometimes,” “always/almost always,” “I don’t know,” or “not relevant.”

For the purposes of this paper, we focused on questions 3) and 4), dealing with friends and family respectively because individuals typically identify strongly with these groups. Material illustrating the responses to the other questions can be found in the supplementary files (S1–S4 Figs).

An open-ended question gave respondents the opportunity to articulate their attitudes, feelings, and experiences regarding protective mask-wearing during the pandemic: “How would you describe your experience with mask-wearing during the pandemic?” In order to encourage more complete responses, we specified the question further: “Did wearing a mask trigger any particular feelings for you? (For example, did wearing a mask annoy or embarrass you? Did you feel safer when wearing a mask? Did you feel a sense of solidarity with others who wore a mask?) Please explain your experiences, thoughts, and feelings in as much detail as possible.” Participants agreed at the outset of the survey to answer open-ended questions, but some responses (n = 25) were excluded from the qualitative analysis due to nonsensical content (i.e., random typing of nonsensical repetitions to fill the minimum word requirement).

The questionnaire was evaluated for content and reliability by a panel of ten experts during a piloting phase. It included some additional questions (four questions regarding participants’ motivations to comply with public health measures and two additional open-ended questions) not directly related to the line of questioning pursued in this paper. (See S1 Text for the complete survey.)

### Ethical approval

This study was approved by the Local Psychological Ethics Committee of the University Medical Center Hamburg–Eppendorf (LPEK-0539). Participation was voluntary and followed a double-opt-in process. All individuals provided written informed consent online. No minors were included in the survey.

### Recruiting, participant selection, and timing

The market research firm Bilendi & respondi recruited participants from across Germany through its own online access panel. The company’s quality management fulfills the strict criteria of Norm ISO 20252. Emphasis was placed on intrinsic motivation with only a token monetary incentive of 50 to 75 cents for participation in this survey, which was estimated to take 10–15 minutes (based on 1 Euro per 20 min. rate.)

Individuals aged 18 and above (n = 330) were sampled per online survey between the 10^th^ and 16^th^ of February 2023. This time period allowed us to capture the manner in which views and experiences had solidified over the course of the pandemic. (See the discussion below for a further treatment of this point.) We excluded those under 18, as well as those who did not agree at the outset to answer the open-ended questions with approximately 3–4 sentences.

Another criterion for inclusion was based on participants’ response to a question about hypothetical voting intentions. As mentioned above, we used hypothetical voting intentions as a marker for political party affiliation due to the fact that only a small percentage (1.66%) of Germans officially belong to any political party. Survey participants were asked how they would vote if elections were to be held the coming Sunday, i.e., the standard “Sunday question.” Participant selection proceeded according to a quota-based sampling designed to include an even number of members who identified with each of the six main political parties in Germany: The Left Party (Die Linke), The Social Democratic Party (SPD), Alliance 90/The Greens (Bündnis 90/Die Grünen), The Free Democratic Party (FDP), and the Christian Democratic Union (CDU, including its closely related ‘sister party’, the Christian Social Union, CSU). In order to avoid repetition in this paper, these hypothetical voters are sometimes referred to merely as “voters”. Participants were also given the options to respond, “I won’t be voting,” “I’m not eligible to vote,” and “no answer,” and those who chose these responses were excluded. The first 55 respondents who identified with each party and who fulfilled the other two criteria were accepted into the survey. This resulted in a total of n = 330 participants.

### Data analysis

Invalid responses were excluded, leaving the valid responses to form an ordinal scale (“not so much” = 1, “sometimes” = 2, and “always/almost always” = 3), from which an average point value (APV) was derived. We used descriptive statistics to obtain frequency tables showing the percentage participant responses associated with each of the six political parties. Adjusting for age, gender, and income, we performed six multiple ordered logistic regressions for outcomes, setting up each party successively as a reference to be able to compare the supporters of each party against each other. Quantitative data analyses were performed using IBM SPSS Statistics 26. The significance level was set to an alpha level of 0.05 for all analyses. Log-odds and significant variations among group-based responses are reported.

Responses to the open-ended question underwent a pragmatic inductive coding process and were categorized according to the Standards for Reporting Qualitative Research (SRQR) [37]. Two advanced researchers, a psychotherapist with PhD in Psychology and a PhD candidate in Medical Sociology, coded responses and induced prevalent categories and themes, working individually first, then cross-checking their work and developing a category system. In accordance with standard coding procedures, responses that contained elements of more than one category were counted in each category, and responses that were wholly unclear were excluded from the count. A third researcher (a philosopher with a PhD and a focus on political and social theory) then compared the individual category assignments of the two assessors and identified all discrepancies. Where categorizations diverged, differences were discussed by the three researchers until a consensus was reached on final decisions. During the process, the researchers hid respondents’ party affiliations and other participant information to avoid bias. An English native speaker translated the responses cited in this paper and these translations were then cross-checked by a second native speaker. After this process was completed, the number of respondents whose voting intentions aligned with each of the six parties was counted in each category.

## Results

Participants included males (49%) and females (51%). The median age of respondents was 47 years with a range from 18 to 87 years. Income levels were as follows: less than 2.500 Euro: 34,1%; 2.500–4.999 Euro: 47,1%; 5000 or more: 12,4%.; no response: 6,3%.

### Health authorities: Doctors and public health institutes

**Fig 1:**
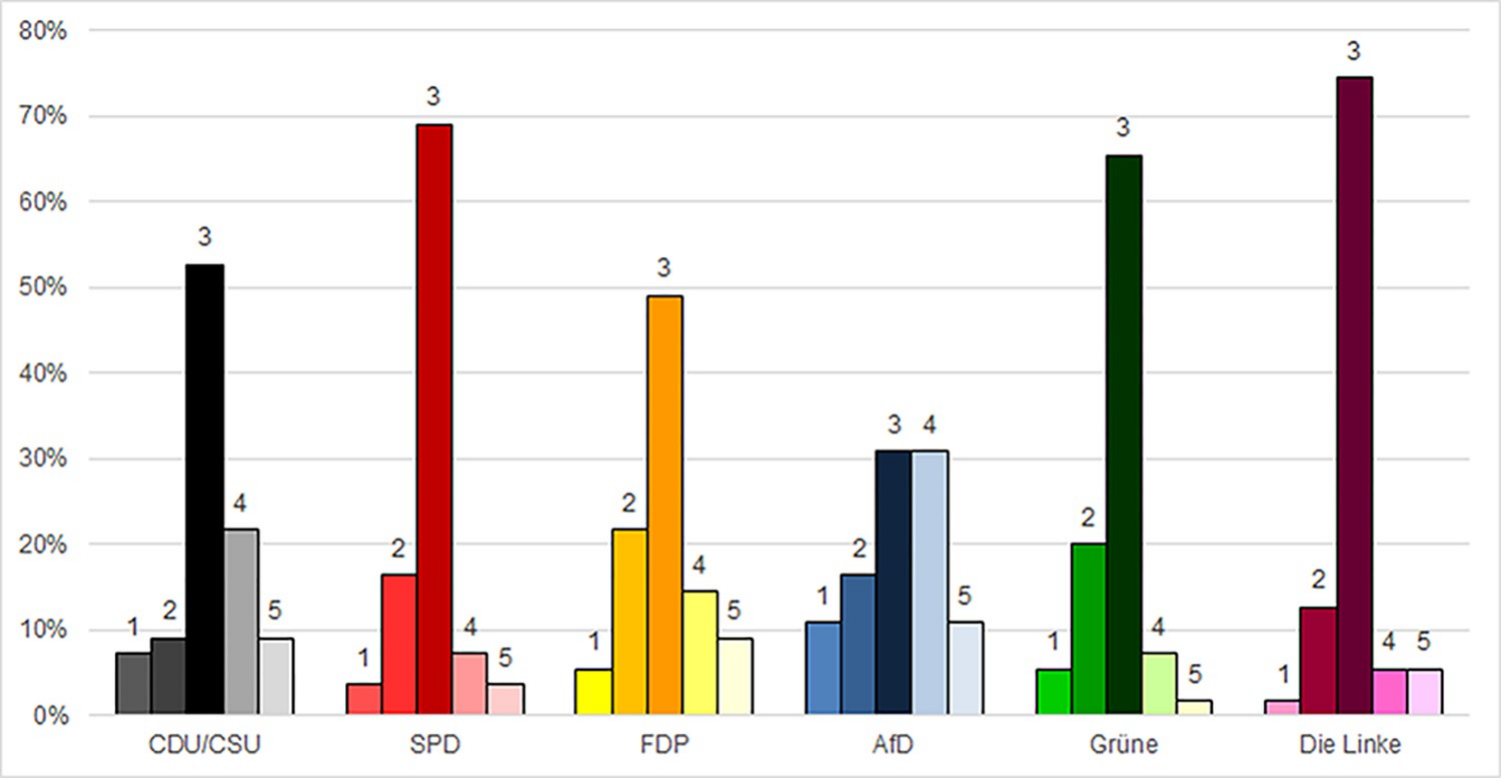
Protective mask-wearing behavior reported to concur with doctors’ advice. 1 = not so much; 2 = sometimes; 3 = always/almost always; 4 = not relevant; 5 = I don’t know.

As Fig 1 shows, approximately 57% of all survey participants responded that their decision to wear a mask–or not to wear a mask–concurred with the advice of their doctor: “always/almost always,” while 16% responded “sometimes” (Fig 1). A majority of Left Party, Green Party, SPD, and CDU/CSU voters responded “always/almost” always, while almost half of FDP voters and slightly over 30% of AfD voters gave this response (Fig 1). SPD and Left Party voters were the most likely to respond that their behavior concurred with their doctors “always/almost always.” (Fig 1).

This figure gives a clear picture of divergences across party lines; however, calculated averages based on a three-point scale allowed a more precise quantification of these differences. These averages showed that AfD voters were least likely to acknowledge a correspondence between their decisions to wear or not to wear a mask and the advice of their doctors with an average point value (APV) of 2.34, while Left Party voters were most likely to do so (APV: 2.82) (S1 Table). Results from ordinal regressions presented in Table 1 showed that AfD voters deviated significantly from all other voter groups except for the FDP. Left Party voters deviated the most significantly (-1.651) from the AfD, while the FDP did not deviate significantly from other groups (Table 1).

**Table 1:** Protective mask-wearing behavior reported to concur with doctor’s advice. Results from ordinal regressions: Log-odds and significance levels.

	AfD	CDU/CSU	SPD	FDP	Left Party	Green Party
AfD	∕	-1.107*	-1.178*	-0.773	-1.651**	-0.956*
CDU/CSU	1.107*	∕	-0.071	0.334	-0.544	0.151
SPD	1.178*	0.071	∕	0.405	-0.473	0.222
FDP	0.773	-0.334	-0.405	∕	-0.878	-0.183
Left Party	1.651**	0.544	0.473	0.878	∕	0.695
Green Party	0.956*	-0.151	-0.222	0.183	-0.695	∕

*p < 0.05.

**p < 0.01.

***p < 0.001.

*Note*: The column on the far left lists the reference party for each regression. n = 330. Invalid responses: “Not relevant”: 48; “I don’t know”: 22.

**Fig 2:**
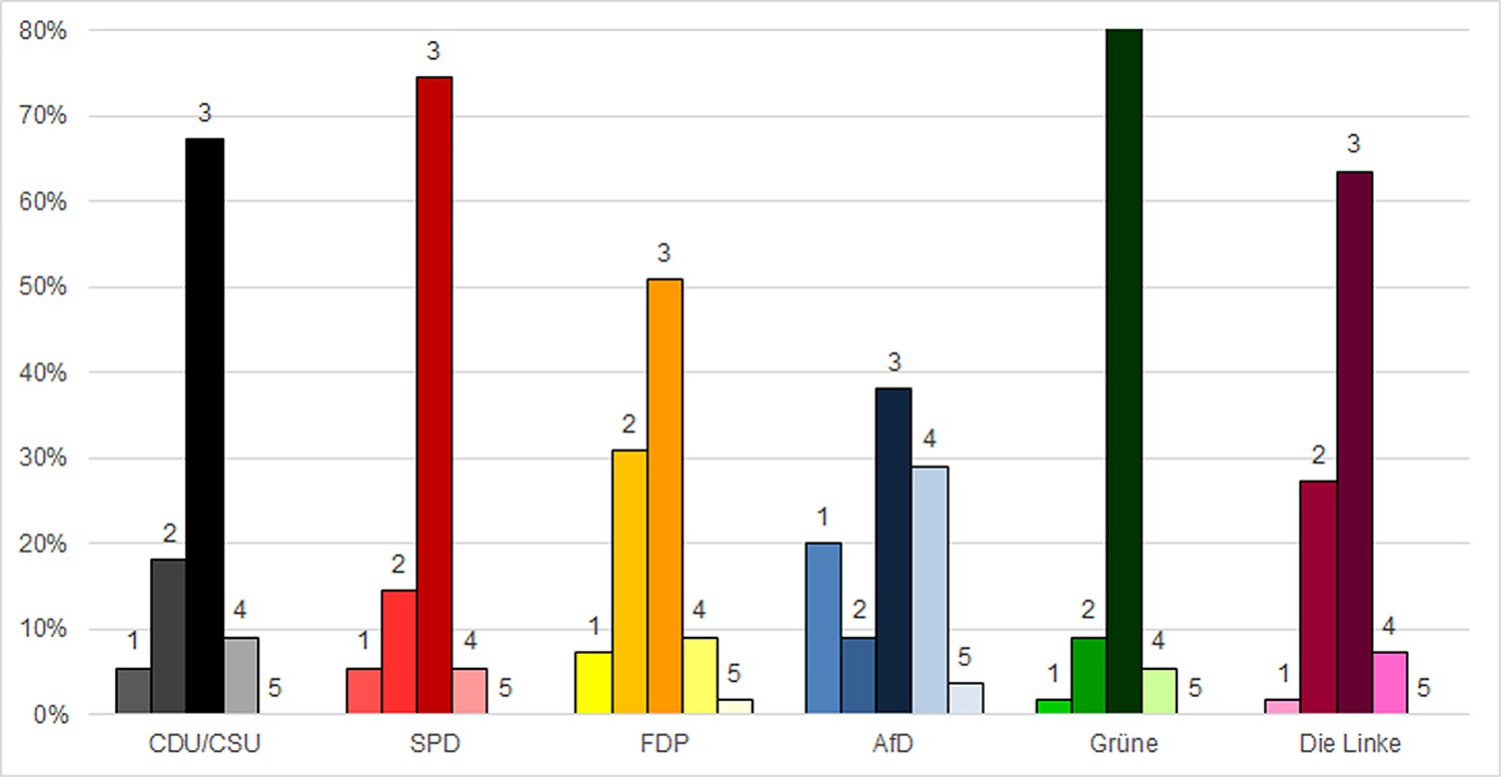
Protective mask-wearing behavior reported to concur with the advice of public health institutes. 1 = not so much; 2 = sometimes; 3 = always/almost always; 4 = not relevant; 5 = I don’t know.

As Fig 2 shows, 63% of survey participants responded that their decision to wear a mask–or not to wear a mask—concurred with the advice of public health institutes such as the Robert Koch Institute “always/almost always,” while 18.2% responded “sometimes” to this question (Fig 2). More than 60% of survey participants from the Green Party, the Left Party, SPD, and CDU/CSU reported that their behavior concurred with the advice of public health institutes “always/almost always.” The less than 40% of AfD voters who responded “always/almost always” contrasts starkly with the 80% of Green Party voters who responded this way (Fig 2).

Average point values based on the weighted three-point scale determined that Green Party voters were the most compliant with the advice of health institutions (APV: 2.87), while AfD voters to be the least attentive to this advice (APV: 2.27) (S1 Table). As Table 2 shows, all groups except the FDP voters deviated significantly from AfD voters. The Greens deviated the most significantly (-2.165) from AfD voters, followed by the SPD (-1.306), the CDU/CSU (-1.061) and Left Party (-0.957). Green Party voters also deviated significantly from the CDU/CSU, Left Party, and FDP, but not from the SPD. The responses of FDP voters were positioned between the AfD and the other parties’ values, deviating significantly only from the Greens (Table 2).

**Table 2:** Protective mask-wearing behavior reported to concur with the recommendations of public health institutes. Results from ordinal regressions: Log-odds and significance levels.

	AfD	CDU/CSU	SPD	FDP	Left Party	Green Party
AfD	∕	-1.061*	-1.306**	-0.564	-0.957*	-2.165***
CDU/CSU	1.061*	∕	-0.245	0.498	0.105	-1.103*
SPD	1.306**	0.245	∕	0.742	0.349	-0.858
FDP	0.564	-0.498	-0.742	∕	-0.393	-1.601**
Left Party	0.957*	-0.105	-0.349	0.393	∕	-1.208*
Green Party	2.165***	1.103*	0.858	1.601**	1.208*	∕

*p < 0.05.

**p < 0.01.

***p < 0.001.

*Note*: The column on the far left lists the reference party for each regression.

n = 330. Invalid responses: “Not relevant”: 36; “I don’t know”: 3.

With regard to both of these questions pertaining to health authorities, AfD voters were the least attentive to these authorities, followed by the FDP.

### The behavior of friends and family

**Fig 3:**
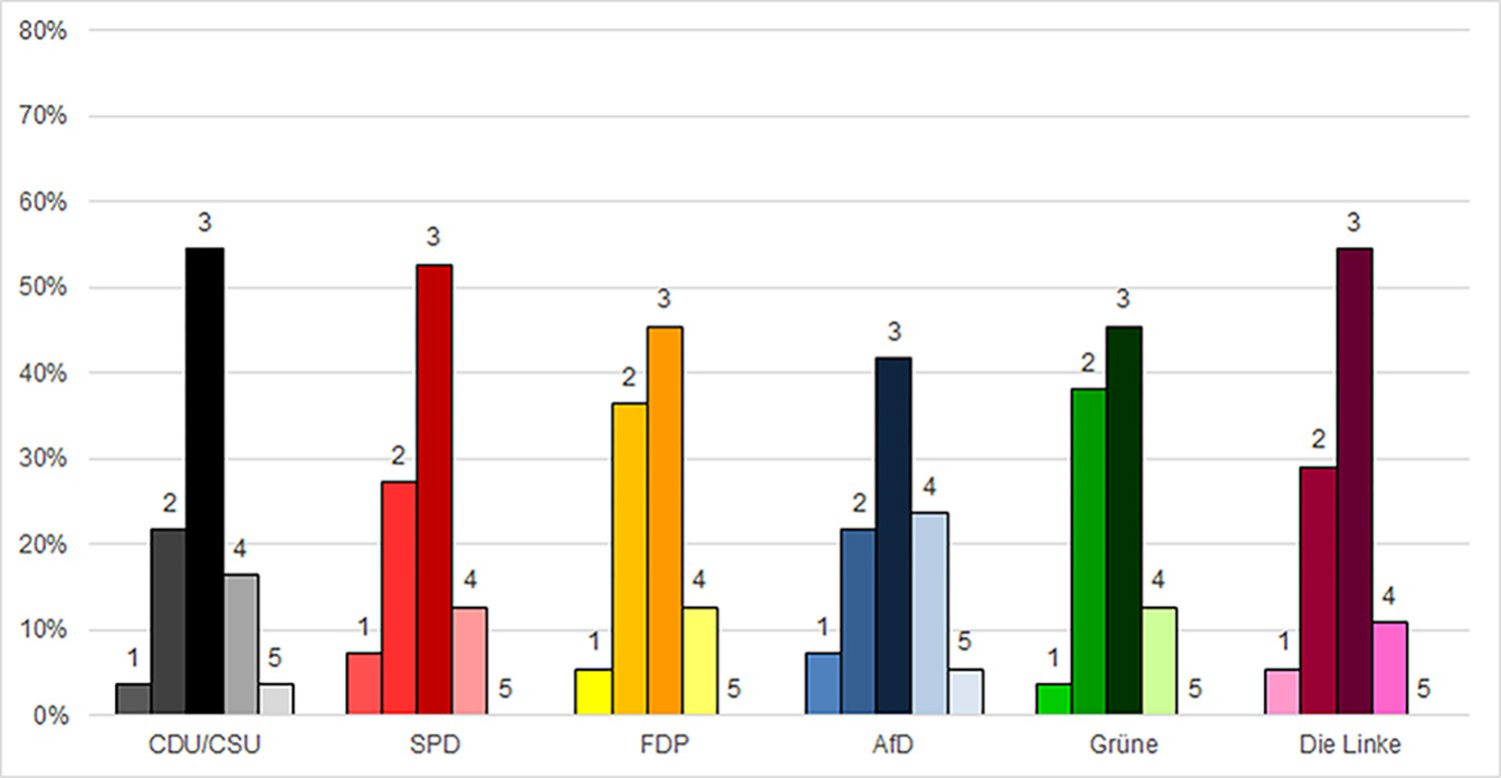
Protective mask-wearing behavior reported to concur with behavior of friends and acquaintances. 1 = not so much; 2 = sometimes; 3 = always/almost always; 4 = not relevant; 5 = I don’t know.

Approximately 49% of all survey participants responded that their mask-wearing behavior concurred with that of their friends and acquaintances "always/almost always," while another 29% responded “sometimes” (Fig 3). Always/almost responses ranged from 41% of AfD voters to 54.5% of CDU/CSU, with the Left party voters, with SPD, FDP, and the Green Party all ranging between 45.4% and 52.7%. At least 20% of voters affiliated with each party said that their mask-wearing behavior concurred with that of their friends and acquaintances “sometimes.” Almost 40% of FDP and Green Party voters responded this way (Fig 3).

Averages of the three-point scale were relatively high across all parties. Results ranged from an APV of 2.46 (FDP) to an APV of 2.64 (CDU/CSU) (S1 Table). As Table 3 shows, there were no significant deviations associated with party preference.

**Table 3:** Protective mask-wearing behavior reported to concur with behavior of friends and acquaintances. Results from ordinal regressions: Log-odds and significance levels.

	AfD	CDU/CSU	SPD	FDP	Left Party	Green Party
AfD	∕	-0.440	-0.069	0.147	-0.172	0.134
CDU/CSU	0.440	/	0.371	0.587	0.268	0.574
SPD	0.069	-0.371	/	0.217	-0.102	0.203
FDP	-0.147	-0.587	-0.217	/	-0.319	-0.014
Left Party	0.172	-0.268	0.102	0.319	/	0.305
Green Party	-0.134	-0.574	-0.203	0.014	-0.305	/

*p < 0.05.

**p < 0.01.

***p < 0.001.

*Note*: The column on the far left lists the reference party for each regression.

n = 330. Invalid Responses: 49 (not relevant); 5 (I don’t know).

**Fig 4:**
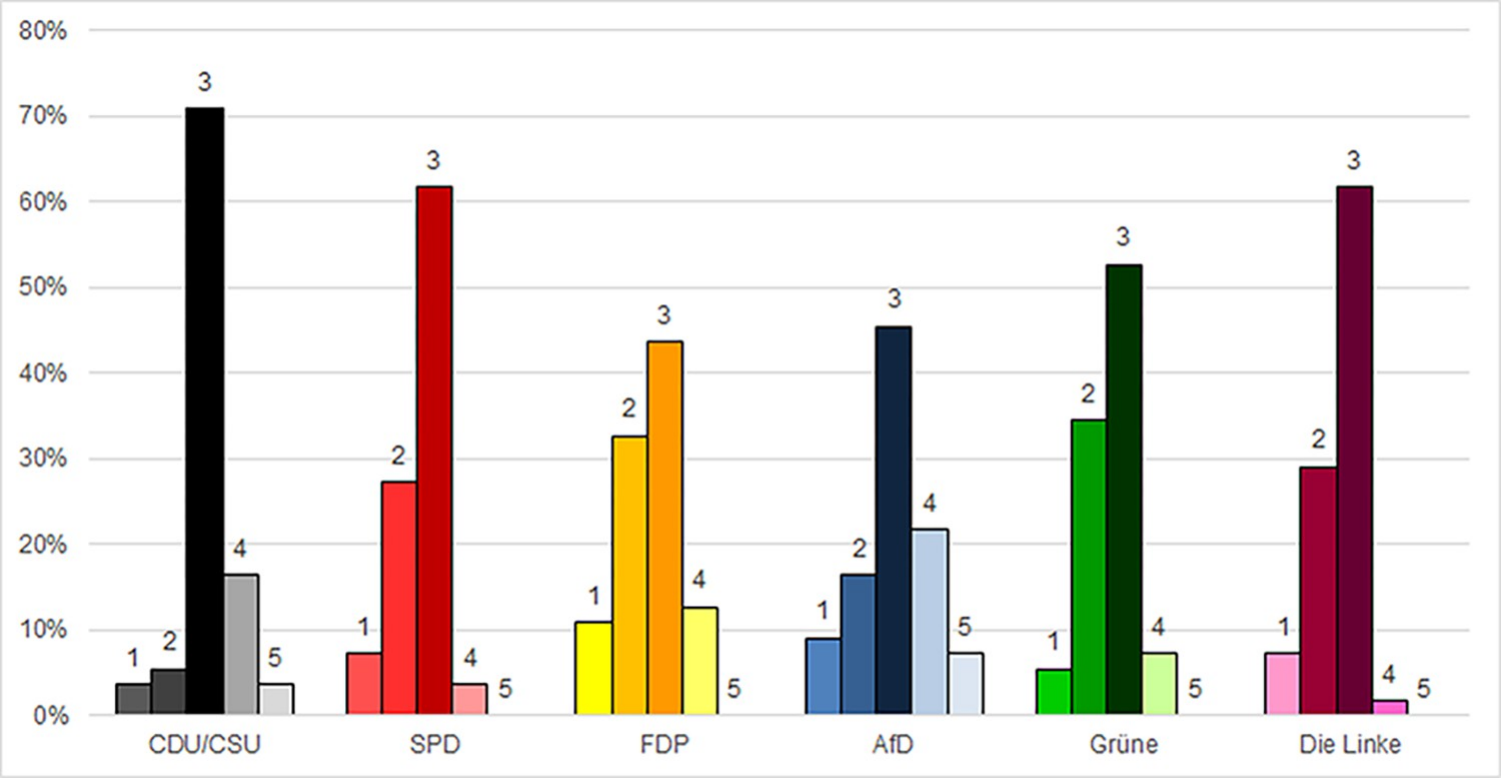
Protective mask-wearing behavior reported to concur with behavior of family members. 1 = not so much; 2 = sometimes; 3 = always/almost always; 4 = not relevant; 5 = I don’t know.

Among participants, the acknowledgement of one’s own behavior as concurring with family members was slightly higher than with friends. Altogether approximately 56% of survey participants responded always/almost always,” while 24% responded “sometimes” (Fig 4). Broken down according to party, 45.5% of AfD voters responded “always/almost always,” marking the lower end, while 70.9% of CDU/CSU comprised the higher end. Prospective CDU/CSU voters stood out as the party most likely to align their behavior with that of family members (Fig 4).

Average point values calculated from the weighted scale ranged between 2.38 (FDP) and 2.84 (CDU) (S1 Table). Multiple regression analyses demonstrated that CDU/CSU voters deviated significantly from all other parties (Table 4). No other significant deviations were found.

**Table 4:** Protective mask-wearing behavior reported to concur with family members. Results from ordinal regressions: Log-odds and significance levels.

	AfD	CDU	SPD	FDP	Left Party	Green Party
AfD	∕	-1.293*	-0.081	0.484	0.071	0.280
CDU	1.293*	∕	1.374**	1.777***	1.364*	1.573**
SPD	0.081	-1.374**	∕	0.402	-0.011	0.198
FDP	-0.484	-1.777***	-0.402	∕	-0.413	-0.204
Left Party	0.071	-1.364*	0.011	0.413	∕	0.209
Green Party	-0.280	-1.573**	-0.198	0.204	-0.209	∕

*p < 0.05.

**p < 0.01.

***p < 0.001.

*Note*: The column on the far left lists the reference party for each regression.

n = 330. Invalid responses: “Not relevant”: 35; “I don’t know”: 6.

In contrast to the relatively high percentage of survey participants who said that their mask-wearing behavior aligned at least “sometimes” with that of their family and friends, relatively few described their behavior as aligning with others who shared political affiliations and views. Overall, 43.2% of all respondents reported that their behavior aligned at least sometimes with the behavior of those from the same political party (S3 Fig). 60.9% reported that their behavior aligned at least sometimes with those who share their political views (S4 Fig).

Another noteworthy result was the high number of participants who responded, “not relevant” to many of the questions (Figs 1–4 and S1–S4). In accordance with standard practice, these responses were considered invalid and excluded from the quantitative analyses presented above. However, the fact that they were so prevalent–especially among voters of certain parties–suggested the need for further consideration.

A clear pattern emerged in that AfD voters were the most likely to respond “not relevant” to each of the questions. In all cases but one, they were followed by CDU/CSU voters, sometimes relatively closely, sometimes at a considerable distance. For example, to the question asking about their adherence to the advice of their doctors, 30.9% of AfD voters and 21.8% of CDU/CSU voters responded that the question was irrelevant (Fig 1). When asked about RKI recommendations, close to 30% of AfD voters also said the question was irrelevant, while less than 10% of any of the other groups responded this way (Fig 2).

A similar pattern occurred in responses to most of the other questions as well (S1–S4 Figs). In each case AfD voters answered “not relevant” more frequently than voters from any of the other parties (Table 5).

**Table 5:** Percentage of respondents answering “not relevant” to questions 3–8.

	AfD	CDU	FDP	Green Party	Left Party	SPD
3. Friends and acquaintances	23.6%	16.4%	12.7%	12.7%	10.9%	12.7%
4. Family and relatives	21.8%	16.4%	12.7%	7.3%	1.8%	3.6%
5. Colleagues	25.5%	21.8%	12.7%	10.9%	18.2%	16.4%
6. Community groups	34.5%	27.3%	14.5%	16.1%	21.8%	16.4%
7. Members of political party	38.2%	27.3%	23.6%	32.1%	21.8%	21.8%
8. Same political views	30.9%	18.2%	5.5%	20.0%	16.4%	14.5%

In all of these cases, prospective CDU/CSU voters were the second most likely to respond that the question was irrelevant. Only in the case of question 8 regarding those with similar political views, did the Green Party surpass the CDU/CSU as the voting group with the second most frequent “not relevant” responses. Here 20% of Green party voters responded “not relevant”–still significantly less than the percentage of AfD voters (over 30%).

Given that the survey questions were designed to determine what factors were relevant to voters’ health behavior decisions, “not relevant” responses may well indicate something significant. When asked, for example, whether their decisions to wear a mask concurred with the advice of the RKI, those who responded “not relevant” may have been suggesting not merely that the survey question was irrelevant to them, but also that the RKI’s recommendation did not factor into their decision. A “not relevant” response could, for example, indicate that the respondent never heard of the RKI, had no access to its protocols, or simply did not bother to seek out the RKI’s advice–all relevant indications about their health behavior decisions.

Since our survey included a set of open-ended questions, which allowed participants to discuss their experiences of wearing (or not wearing) masks during the pandemic, we were able to attain some additional context through which to interpret these “not relevant” responses (see the results of the qualitative data below). Preliminarily, we may note here that of the 55 AfD-affiliated respondents, 16 checked “not relevant” three or more times. When we identified the responses to the open-ended questions given by these 16 participants, we found that 10 of these included strongly negative comments about masks and/or mask-mandates. The situation was different with respect to the case of respondents affiliated with the CDU. Sixteen of the 55 CDU/CSU respondents also included three or more “not relevant” responses. However, in the open-ended question, most of these expressed positive views of masks (despite some complaints), with only 3 of the 16 expressing strongly negative ones. The divergence in the content of these responses suggests the need to consider the open-ended responses in greater detail.

### Results of the qualitative data

Seven broad categories with several sub-themes derived from coding the open-ended responses (Table 6).

**Table 6:** Qualitative evaluation of the free text data on experiences, thoughts, and feelings with mask-wearing during the pandemic*.

Categories	Key aspects	Exemplary responses	According to affiliation	
Safety			**Total n = 178**	
	self-protection, protection of others	“I felt much safer with the mask….the fact that other people wore masks strengthened my feeling of security”	CDU /CSU	n = 39
			Green Party	n = 37
			SPD	n = 33
			Left Party	n = 28
			AfD	n = 21
			FDP	n = 20
Problem, but acceptance			**Total n = 129**	
	basic acceptance despite difficulties	“Although I, as an asthmatic, had problems wearing [the masks], I persevered….”	FDP	n = 28
			Left Party	n = 26
			SPD	n = 23
			CDU /CSU	n = 21
			Green Party	n = 17
			AfD	n = 14
No problem/ got used to it			**Total n = 86**	
	no or small problems with fast or easy assimilation	“I felt better with a mask.” “…with time, you got used to wearing a mask.”	Green Party	n = 18
			CDU/CSU	n = 17
			SPD	n = 16
			AfD and FDP	n = 13
			Left Party	n = 9
Anger/ Annoyance			**Total n = 58**	
	coercion, loss of freedom, uselessness of masks	“I found it idiotic from the beginning. Today it has been confirmed how stupid it all was.”	AfD	n = 24
			FDP	n = 13
			Left Party	n = 12
			Green Party, SPD	n = 4
			CDU/CSU	n = 1
Solidarity			**Total = 46**	
	responsibility for society, public good, social duty	“I felt complete solidarity with other mask-wearers.” “… it strengthened my connection with those around me.”	Green Party	n = 13
			SPD	n = 9
			Left Party	n = 7
			AfD and CDU/CSU	n = 6
			FDP	n = 5
Necessity			**Total = 45**	
	reasonable, self-evident, the only way	“[mask-wearing] was indispensable….”, “What has to be, has to be!”	Left Party	n = 11
			CDU /CSU	n = 10
			FDP	n = 8
			Green Party	n = 7
			SPD	n = 6
			AfD	n = 3
Anger over "refusers“			**Total = 32**	
	Upset at those who didn’t wear masks or wore them incorrectly	“People who didn’t wear a mask are in my opinion ignorant idiots.”	Green Party	n = 11
			CDU /CSU	n = 6
			SPD	n = 5
			FDP and Left Party	n = 4
			AfD	n = 2

*Note: The analysis of the free text responses refers to the following voter groups: FDP: n = 53; AFD: n = 52; CDU/CSU: n = 52; Left Party: n = 50; Green Party: n = 49; SPD: n = 49.

As Table 6 shows, responses were most likely to mention that wearing a mask made them feel safer (n = 178). This was particularly prevalent among Green Party, CDU, and SPD voters, but the experience was reflected across the board. Interestingly, many respondents linked the sense of safety they felt for themselves with the importance of protecting others. For example, one FDP voter noted feeling safer wearing a mask, adding, “Moreover, I found it a good idea to protect others this way.” Similarly, a Green Party voter explained, “I felt safer and was not at all annoyed. Above all, I had the feeling that if I was infected, the virus would not be easily transmitted [to others].” In all parties except the AfD, the sense of security outweighed feelings of anger, resentment, and annoyance. Nonetheless, a good portion of AfD voters expressed positive or neutral views about masks, as several of their responses mentioned either increased safety (n = 21) or indicated little (n = 14) or no (n = 13) problem accepting mask-wearing. As one AfD voter put it, “One felt safer and that is not bad.”

Among voters from all parties, this sense of safety was more frequently mentioned than feelings of solidarity. However, a considerable number of responses mentioned or implied that they had experienced a sense of solidarity with other mask-wearers (n = 46). Green Party voters were the most likely to mention solidarity, but respondents linked to each of the six political parties mentioned (or implied) experiencing a sense of solidarity. Here, one AfD voter noted “a sense of community spirit,” while a CDU/CSU voter alluded to “the common good.” Others referred to a sense of shared purpose (“I felt a sense of solidarity, so that we could end the crisis quickly”–Green Party voter). Some respondents emphasized that the feeling of solidarity depended upon everyone following the mask mandates (“… Besides, everyone wore a mask, that way it wasn’t so bad. One showed solidarity with the others”–Left Party voter). Interestingly, many respondents expressed solidarity not only with other mask-wearers, but also with those suffering from the virus or at high risk for severe consequences from it (“I had the feeling I was helping others when I wore a mask. I thought that I was showing solidarity with those who are sick when I wore a mask”–FDP voter). Others explicitly mentioned a feeling of solidarity with caretakers and medical professionals (“I felt a feeling of solidarity especially with caretakers who had to work on the front lines….”–Green Party voter).

Numerous responses referenced the necessity of mask-wearing (n = 45)–sometimes expressing an understanding of their protective value (“I considered masks an absolute necessity. Wearing them was and is a matter of course….”–CDU/CSU voter), and sometimes expressing their duty to follow the law (“… I felt neither more secure nor especially in solidarity with others. It was the regulation and I always followed it.”–FDP voter). Some, including one Left Party voter and one CDU/CSU voter, spoke of the mask mandate as a “necessary evil.” Most commonly, the necessity of wearing a mask was expressed in a relatively straightforward, unemotional manner (“I wore the mask because it was reasonable and necessary.”–Left Party voter.)

A large number of responses described mask-wearing as unproblematic (n = 86), though some of these mentioned needing a period of adjustment. (“Yes, in the beginning it was embarrassing, but with time, I got so used to it….”–Green Party voter). Other responses expressed considerable discomfort with masks (n = 129), but nonetheless accepted them. Problems frequently mentioned were difficulty breathing, difficulty communicating, and foggy glasses. For example:

“Sometimes I had problems breathing through the mask. But otherwise it was ok….” (AfD voter)

“The mask was ok for me. However, due to a chronic lung disease, I could not breathe as well.” (CDU/CSU voter)

“It was in fact unpleasant and unusual, especially as an eyeglass-wearer, my glasses fogged up regularly; however, I felt it was right and important [to wear a mask].” (Green Party voter)

“It was necessary, but I suffered shortness of breath….” (Left Party voter)

“…. It was strange. Especially because one could not see the facial expressions of others….” (FDP voter)

A large share of the responses, especially from prospective AFD voters, were dominated by negative feelings such as anger, resentment, and extreme annoyance (n = 58). Many of these respondents connected their annoyance and anger with the feeling or belief that mask regulations infringed on their freedom. As one AfD voter exclaimed: “I felt annoyed, fooled, and like a slave!” Another wrote, “… I found [wearing a mask] super disturbing.… And no, I didn’t feel solidarity… rather I was shocked to see how all the well-behaved people, like a herd of mutton, just followed the leader without questioning” (AfD voter). Several survey participants, especially some potential AfD voters, expressed the belief that protective masks were ineffective and even harmful: “Masks don’t do anything. Everyone should have realized that by now. Masks serve as a sign of suppression of the population” (AfD voter). Another commented, “It was clear from the beginning that these masks do nothing. Despite this, mask-wearing was pushed come hell or high water. I consider these masks to be burdensome and unhealthy….” (AfD voter).

Some voters affiliated with the other parties also expressed significant annoyance with masks, although very few of them complained about a loss of freedom. In general, their comments tended to be less vehement, and they highlighted other, perhaps less serious concerns. Respondents from several parties, for example, noted how annoying it was when they forgot their mask on the way somewhere and had to go all the way back home to get it. Others emphasized the lack of hygiene involved in what they considered typical mask use. As one Green Party voter complained, “The masks were never changed, and you could see how dirty they were. They offered no protection like that.” An FDP voter made a similar observation, while also emphasizing the alienating effect of masks: “I found mask-wearing alienating and extremely unhygienic… The majority did not know how to use them properly, and I think that masks in the end did more harm than good. People seemed less human and mask politics was more an against-each-other than a for-or-with another.” Another factor that led to frustration was the inconsistency in how mask mandates were enforced. One Left Party voter explained having no problem with masks at first, but becoming increasingly frustrated with masks as time went on: “… certain things like corona-denier demonstrations were allowed, while I, for example, as a worker in a small business carrying heavy crates, had to wear an FFP2 mask even before the store opened.”

A final theme that came up repeatedly, especially among Green Party voters, was anger and annoyance at those who refused to wear masks or who wore them the wrong way. Typical responses were as follows:

“… People who wear the masks under their noses make me furious.” (Green Party voter)

“… What bothered me was people who wore the mask incorrectly or as kind of alibi…” (Left Party voter)

An FDP voter noted an “unpleasant feeling” whenever someone failed to wear a mask.

## Discussion

Our quantitative and qualitative results indicated that participants’ adherence to health professionals’ guidelines regarding protective mask-wearing varied according to their political party affiliations. In particular, AfD supporters were the least inclined to heed such advice and the most likely to express negative views and feelings about protective mask-wearing. Green Party voters were most likely to consider public health regulations, and Left Party members were most likely to consider the advice of their doctors. Behavioral cues provided by members of shared social groups, such as family and friends, played an important role across party lines. Our analysis thus lends weight to the position that supporters of Germany’s far-right party were less compliant and more resistant to at least some of the COVID-19 public health measures than affiliates of other political parties in Germany. (See the introduction to this paper). At the same time, our study challenges the common belief that populists on both ends of the political spectrum were equally resistant to the recommendations of health authorities, as it revealed considerable differences between Germany’s left and right political parties in this regard.

Although our study indicated that political party affiliations may have influenced mask-wearing behavior in Germany during the pandemic, it did not produce evidence for the extreme forms of polarization found in countries like the USA and Brazil, where the wearing (or not wearing) of masks became highly politicized and emotionally charged [38,39]. More studies will need to be conducted in order to determine the reasons for this. However, it is worth noting that this lack of extreme polarization can be explained, to some degree, by SIA principles. Although German citizen’s willingness to vote for a given party establishes a degree of identification with that party, SIA principles suggest that this form of group identification will be more fluid than identifications that occur when individuals see themselves explicitly as group members [40]. It is therefore not surprising if, in comparison with voters from other countries who understand their party membership as fundamental to their identity, German citizens may be less susceptible to “in group” pressures to support the entire political agenda of any one political party. SIA principles also show that the tendency of individuals to fall into an “us-them” mentality may be mitigated by a more complex set of choices [41]. To some extent, then, the existence of several major political parties in Germany may reduce the risk of rigid political polarization, even though a multi-party system does not guarantee protection against political extremism. In addition, the relatively high importance placed on family, friends, colleagues, and other social groups in Germany may play a role in reducing political polarization, for, as our study also suggests, German citizens may be more inclined to orientate themselves toward these social groups than toward political groups [Figs 1–4 and S1–S4].

Our study operated within a methodological framework that had both strengths and limitations. As is the case with any study based on a survey, our study was limited by its reliance on the self-reporting of individuals, who may not have truthfully reported their behavior [42] or who may have overestimated the independence of their decisions and/or misjudged the influence of external factors on their behavior [43]. Furthermore, because some of our multiple-choice questions were of a partially repetitive nature, some respondents may have paid less attention when responding to later questions. The limited size and non-representative nature of our sample should also be taken into account. In particular, the results of our logistic regressions should be viewed with caution since a sample size of at least 500 is generally recommended to avoid underpowered or biased results. It is important to note, however, that the literature on the required sample size remains inconclusive, with some research suggesting a minimum size lower than 300 [44]. Furthermore, since most sample size calculations assume a more or less random sampling procedure, it is not clear whether or to what extent standard sample size calculations should be applied to quota-based samples. Our quota-based sampling strategy ensured that we had the same number of group observations for our predictor of interest (political party affiliation), thus incorporating a constancy not taken into account by standard sample size calculations.

It is also unclear whether standard sample size calculations designed for purely quantitative studies should be applied to mixed-methods studies such as this one. Qualitative studies necessitate smaller sample sizes than quantitative studies, but they compensate for this “lack” by yielding more in-depth results. We view the inclusion of a qualitative analysis to be an important strength of our study, as it provided a more thorough explanation for our quantitative findings. For this reason, we emphasize the need to interpret our quantitative results in conjunction with the results of our qualitative analysis.

By granting survey participants space to describe their experiences, express their feelings, and develop their views, our inclusion of an open-ended question gave participants the chance to name and pursue topics beyond what was directly specified in the standardized multiple-choice responses. For example, our survey questions did not explicitly distinguish among various kinds of masks. However, the open-ended questions allowed respondents to make these distinctions if they found them relevant, and some respondents took the opportunity to do so. Similarly, none of the questions we included explicitly addressed issues of trust and misinformation, although these issues are closely related to COVID-19-related health behavior [45]. These topics were beyond the explicit scope of our study, but our open-ended questions gave respondents the chance to address them if and when they felt they were relevant. Surprisingly, given the importance of the topic, relatively few respondents thematized these issues [Table 6]. Although personal interviews would have had the advantage of allowing for follow-up questions, the increasing volatility that has come to surround questions pertaining to political party affiliation (especially with regard to radical right parties) led us to opt for an online survey with an open-ended question instead. This had the compelling advantage of allowing participants to remain anonymous. Since some interviewees adjust their responses out of fear of the interviewer’s implicit judgement–especially when sensitive topics are involved–we considered this anonymity crucial to ensuring an environment wherein individuals felt free to express their real views [46]. In this way, we were able to obtain detailed responses to an open-ended question, which provided clarification and context to the multiple-choice responses. However, the results of our qualitative analysis should also be interpreted with caution due to the inherently subjective nature of qualitative analyses and due to the fact that some respondents gave more detailed answers than others. These results are meant to be merely illustrative and should not be interpreted to indicate any causal connections. Nonetheless, when taken together with our quantitative results, they suggest political and social tendencies worthy of further study.

This study is one of the first to document how views about mask-wearing coalesced over the pandemic’s approximately three-year period. In contrast to previous studies which focused on a particular phase of the pandemic or documented shifts in attitudes and behavior throughout the course of the pandemic, our survey took place shortly after the German government dropped mask mandates on long-distance trains, a move widely taken to signify the end of the pandemic as a major public health threat [47]. Since we asked participants to recall the early phases of the pandemic in particular, our study suffers under the limitations of participants’ memories. However, by conducting our survey during February 2023, we were able to gain insight into how individuals sized up their entire experience of masks during the pandemic. This is important not only because the views and feelings about masks shifted over time, but also because they are likely to have *evolved* in a particular direction, depending upon individuals’ experiences during the pandemic. For example, the cumulative experience of three years of mask requirements may have resulted in a “fatigue” that will reduce some people’s willingness to comply with mask requirements in the event of another pandemic. Conversely, the experience of loss resulting from a lack of compliance may lead some people to be more vigilant the next time. Either way, the views and feelings that citizens were left with at the end of the pandemic are likely to form the basis of their future response to mask regulations should they become necessary again in the future.

In addition to producing results about German citizens in particular, our study highlights the ways in which political affiliations can both encourage and discourage health compliance. As other social identity theorists have observed, a shared sense of identity may increase a sense of social unity and promote compliance with public health measures [48], but it may just as easily exacerbate social differences, set off reactive stances, and discourage compliance with such measures [49,50]. Further studies are needed to determine exactly which group identifications and circumstances lead to social bonds that encourage a sense of the common good and public health compliance, on the one hand, and which ones foster reactive stances, social divisions, and lower compliance levels, on the other. Our study suggests that political party affiliation is an important, if somewhat overlooked, consideration in this regard.

## Supporting information

S1 Fig Protective mask-wearing behavior reported to concur with colleagues. (PDF)

S2 Fig Protective mask-wearing behavior reported to concur with members of shared community groups. (PDF)

S3 Fig Protective mask-wearing behavior reported to concur with members of the same political party. (PDF)

S4 Fig Protective mask-wearing behavior reported to concur with those who share similar political views. (PDF)

S1 Table Average point values (Q1-8) from each of the six main political parties in Germany. (DOCX)

S2 Table Results of ordinal regressions from multiple choice questions 5–8. (DOCX)

S3 Table P values. (PDF)

S1 Text Survey in English translation. (DOCX)
