# Supplementary figures and images for: Political party affiliation, social identity cues, and attitudes about protective mask-wearing during the COVID-19 pandemic in Germany
Source: PLoS One. 2024 Jun 6;19(6):e0302399. doi: 10.1371/journal.pone.0302399 (PMC11156322; doi:10.1371/journal.pone.0302399)

S2 Figure Colleagues

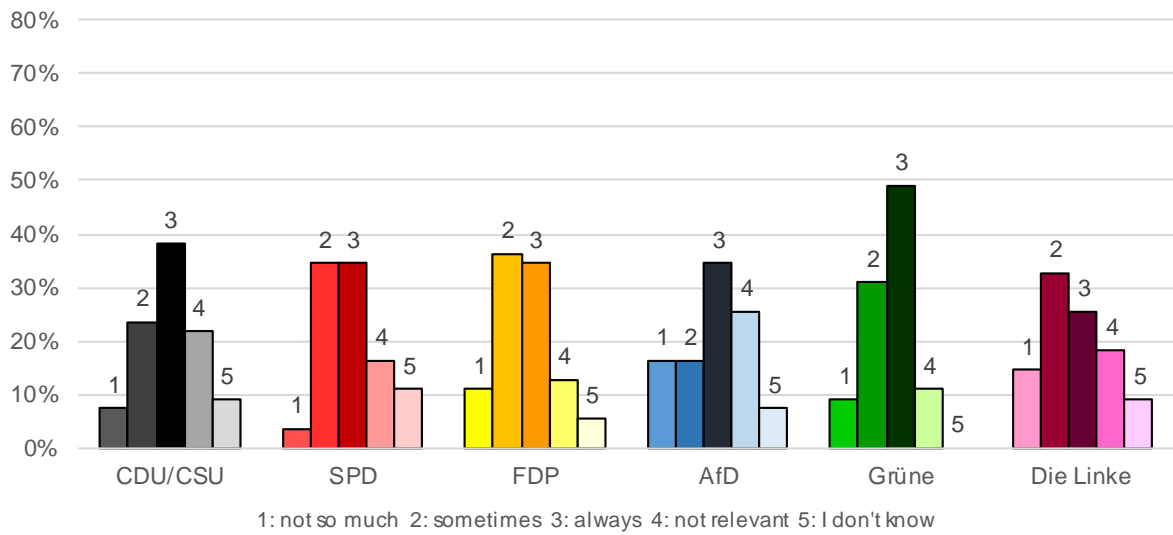

Supplement: S1 Fig — (PDF) [file pone.0302399.s001.pdf]

S3 Figure Community

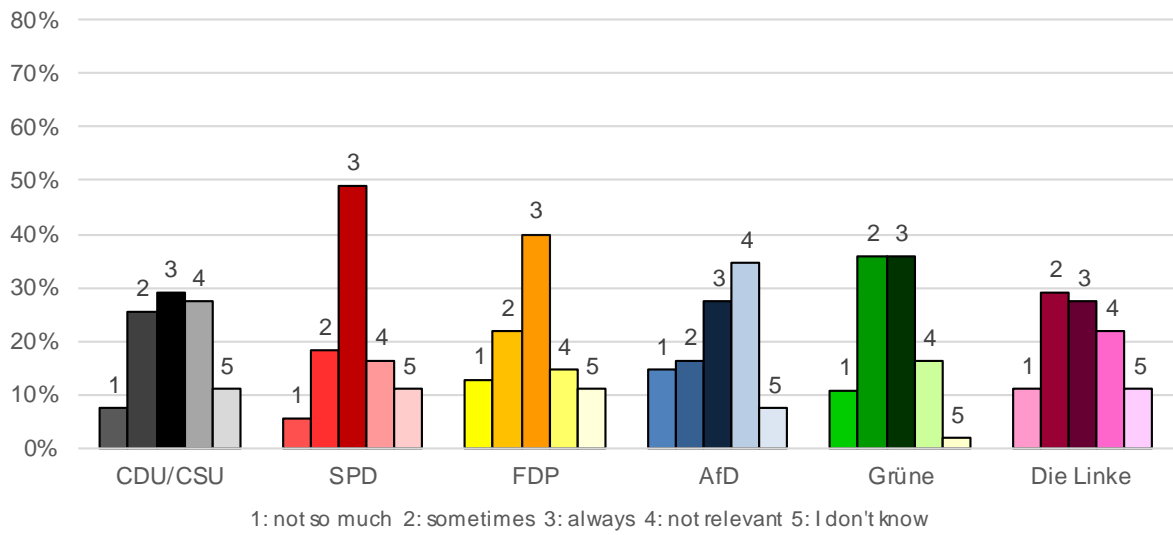

Supplement: S2 Fig — (PDF) [file pone.0302399.s002.pdf]

S4 Figure Party Members

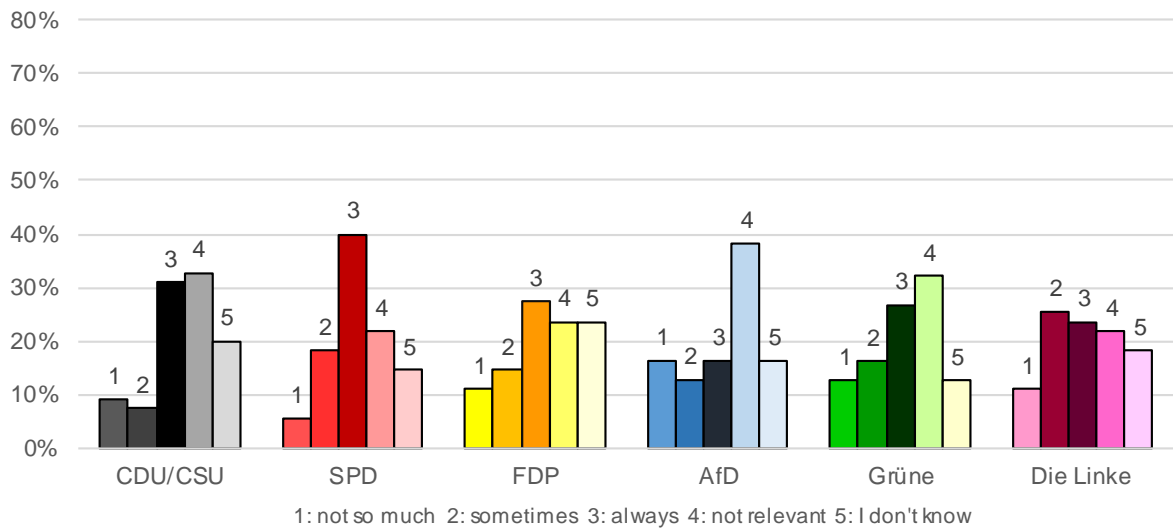

Supplement: S3 Fig — (PDF) [file pone.0302399.s003.pdf]

S5 Figure Political View

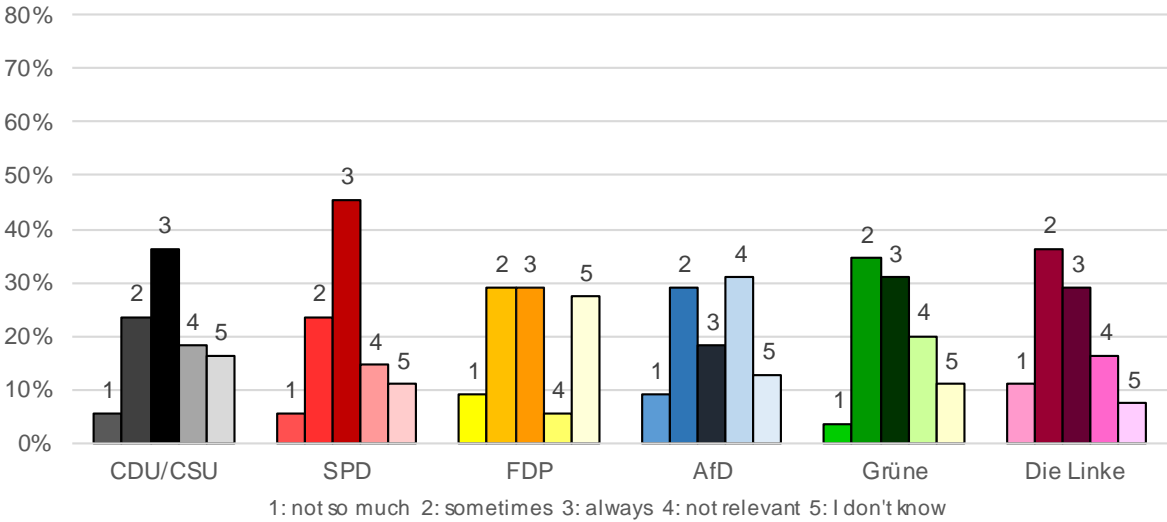

Supplement: S4 Fig — (PDF) [file pone.0302399.s004.pdf]
