# Supplementary material for: Political party affiliation, social identity cues, and attitudes about protective mask-wearing during the COVID-19 pandemic in Germany
Source: PLoS One. 2024 Jun 6;19(6):e0302399. doi: 10.1371/journal.pone.0302399 (PMC11156322; doi:10.1371/journal.pone.0302399)
Supplement: S1 Table — (DOCX) [file pone.0302399.s005.docx]

**S1 Table. Average Point Values (Q1-8) from each of the six main political parties in Germany**

|  | AfD | CDU | FDP | Grüne | Linke | SPD |
| --- | --- | --- | --- | --- | --- | --- |
| 1. Doctors | 2.34 | 2.66 | 2.57 | 2.66 | 2.82 | 2.73 |
| 2. Public health institution | 2.27 | 2.68 | 2.49 | 2.87 | 2.67 | 2.73 |
| 3. Friends and acquaintances | 2.49 | 2.63 | 2.46 | 2.48 | 2.55 | 2.52 |
| 4. Family and relatives | 2.51 | 2.84 | 2.38 | 2.51 | 2.56 | 2.57 |
| 5. Colleagues | 2.27 | 2.44 | 2.29 | 2.45 | 2.15 | 2.43 |
| 6. Community groups | 2.22 | 2.35 | 2.37 | 2.3 | 2.24 | 2.6 |
| 7. Members of political party | 2.0 | 2.46 | 2.31 | 2.26 | 2.21 | 2.54 |
| 8. Same political views | 2.16 | 2.47 | 2.3 | 2.39 | 2.24 | 2.54 |

Rounded to the second decimal place. Rounded up from 0.005.
