# Supplementary material for: Political party affiliation, social identity cues, and attitudes about protective mask-wearing during the COVID-19 pandemic in Germany
Source: PLoS One. 2024 Jun 6;19(6):e0302399. doi: 10.1371/journal.pone.0302399 (PMC11156322; doi:10.1371/journal.pone.0302399)
Supplement: S2 Table — (DOCX) [file pone.0302399.s006.docx]

|  | AfD | CDU | SPD | FDP | Die Linke | Die Grüne |
| --- | --- | --- | --- | --- | --- | --- |
| AfD | / | -0.454 | -0.204 | -0.132 | 0.308 | -0.469 |
| CDU | 0.454 | ∕ | 0.250 | 0.322 | 0.761 | -0.015 |
| SPD | 0.204 | -0.250 | ∕ | 0.072 | 0.512 | -0.265 |
| FDP | 0.132 | -0.322 | -0.072 | ∕ | 0.440 | -0.336 |
| Die Linke | -0.308 | -0.761 | -0.512 | -0.440 | ∕ | -0.776 |
| Die Grüne | 0.469 | 0.015 | 0.265 | 0.336 | 0.776 | ∕ |

**S6 Tables.**

**Results of ordinal regressions from multiple choice questions** **5-8******

Table 9. Q 5. Protective mask-wearing behaviour reported to concur with colleagues

Deviation from reference party

Reference party

*p < 0.05

**p < 0.01

***p < 0.001

**** These are numbered Q1 e-h in the survey (S7 Text).

n = 330.

**Table 10. Q 6. Protective mask-wearing behaviour reported to concur with members of shared community groups**

Deviation from reference party

Reference party

|  | AfD | CDU | SPD | FDP | Die Linke | Die Grüne |
| --- | --- | --- | --- | --- | --- | --- |
| AfD | ∕ | -0.351 | -1.033* | -0.573 | -0.067 | -0.222 |
| CDU | 0.351 | ∕ | -0.682 | -0.222 | 0.283 | 0.128 |
| SPD | 1.033* | 0.682 | ∕ | 0.460 | 0.966* | 0.811 |
| FDP | 0.573 | 0.222 | -0.460 | ∕ | 0.506 | 0.351 |
| Die Linke | 0.067 | -0.283 | -0.966* | -0.506 | ∕ | -0.155 |
| Die Grüne | 0.222 | -0.128 | -0.811 | -0.351 | 0.155 | ∕ |

*p < 0.05

**p < 0.01

***p < 0.001

n = 330.

**Table 11. Q 7. Protective mask-wearing behaviour reported to concur with others from the same political party**

Deviation from reference party

|  | AfD | CDU | SPD | FDP | Die Linke | Die Grüne |
| --- | --- | --- | --- | --- | --- | --- |
| AfD | ∕ | -1.191* | -1.301* | -0.822 | -0.579 | -0.692 |
| CDU | 1.191* | ∕ | -0.110 | 0.369 | 0.612 | 0.499 |
| SPD | 1.301* | 0.110 | ∕ | 0.480 | 0.722 | 0.609 |
| FDP | 0.822 | -0.369 | -0.480 | ∕ | 0.242 | 0.129 |
| Die Linke | 0.579 | -0.612 | -0.722 | -0.242 | ∕ | -0.113 |
| Die Grüne | 0.692 | -0.499 | -0.609 | -0.129 | 0.113 | ∕ |

Reference party

*p < 0.05

**p < 0.01

***p < 0.001

n = 330.

**Table 12. Q 8. Protective mask-wearing behaviour reported to concur with those who share similar political views.**

Deviation from reference party

|  | AfD | CDU | SPD | FDP | Die Linke | Die Grüne |
| --- | --- | --- | --- | --- | --- | --- |
| AfD | ∕ | -0.925 | -1.063* | -0.447 | -0.169 | -0.632 |
| CDU | 0.925 | ∕ | -0.138 | 0.477 | 0.756 | 0.292 |
| SPD | 1.063* | 0.138 | ∕ | 0.615 | 0.894* | 0.431 |
| FDP | 0.447 | -0.477 | -0.615 | ∕ | 0.279 | -0.185 |
| Die Linke | 0.169 | -0.756 | -0.894* | -0.279 | ∕ | -0.464 |
| Die Grüne | 0.632 | -0.292 | -0.431 | 0.185 | 0.464 | ∕ |

Reference party

*p < 0.05

**p < 0.01

***p < 0.001

n = 330.
