# Supplementary material for: Political party affiliation, social identity cues, and attitudes about protective mask-wearing during the COVID-19 pandemic in Germany
Source: PLoS One. 2024 Jun 6;19(6):e0302399. doi: 10.1371/journal.pone.0302399 (PMC11156322; doi:10.1371/journal.pone.0302399)
Supplement: S3 Table — (PDF) [file pone.0302399.s007.pdf]

S8 P-values from the ordinal Regressions

Table Question 1: Doctors

Deviation from reference party

Reference party

	AfD	CDU	SPD	FDP	Die Linke	Die Grüne
AfD	⁄	0.037*	0.017*	0.115	0.002**	0.048*
CDU	0.037*	⁄	0.892	0.521	0.335	0.765
SPD	0.017*	0.892	⁄	0.404	0.374	0.640
FDP	0.115	0.521	0.404	⁄	0.087	0.690
Die Linke	1.651**	0.335	0.374	0.087	⁄	0.171
Die Grüne	0.048*	0.765	0.640	0.690	0.171	⁄

Table Question 2: Public Health Institutions

Deviation from reference party

Reference party

	AfD	CDU	SPD	FDP	Die Linke	Die Grüne
AfD	⁄	0.022*	0.005**	0.195	0.033*	<0.001***
CDU	0.022*	⁄	0.609	0.264	0.820	0.046*
SPD	0.005**	0.609	⁄	0.101	0.450	0.125
FDP	0.195	0.264	0.101	⁄	0.352	0.002**
Die Linke	0.033*	0.820	0.450	0.352	⁄	0.024*
Die Grüne	<0.001***	0.046*	0.125	0.002**	0.024*	⁄

Table Question 3: Friends

Deviation from reference party

Reference party

	AfD	CDU	SPD	FDP	Die Linke	Die Grüne
AfD	/	0.340	0.873	0.732	0.695	0.757
CDU	0.340	/	0.399	0.179	0.546	0.185
SPD	0.873	0.399	/	0.602	0.805	0.618
FDP	0.732	0.179	0.602	/	0.434	0.973
Die Linke	0.695	0.546	0.805	0.434	/	0.451
Die Grüne	0.757	0.185	0.618	0.973	0.451	/

Table Question 4: Family

Deviation from reference party

Reference party

	AfD	CDU	SPD	FDP	Die Linke	Die Grüne
AfD	⁄	0.026*	0.854	0.272	0.873	0.526
CDU	0.026*	⁄	0.014*	0.001***	0.014*	0.005**
SPD	0.854	0.014*	⁄	0.372	0.979	0.626
FDP	0.272	0.001***	0.372	⁄	0.302	0.610
Die Linke	0.873	0.014*	0.979	0.302	⁄	0.601
Die Grüne	0.526	0.005**	0.626	0.610	0.601	⁄

Table Question 5: Colleagues

Deviation from reference party

Reference party

	AfD	CDU	SPD	FDP	Die Linke	Die Grüne
AfD	⁄	0.323	0.645	0.763	0.490	0.276
CDU	0.323	⁄	0.579	0.463	0.091	0.973
SPD	0.645	0.579	⁄	0.868	0.241	0.529
FDP	0.763	0.463	0.868	⁄	0.287	0.403
Die Linke	0.490	0.091	0.241	0.287	⁄	0.059
Die Grüne	0.276	0.973	0.529	0.403	0.059	⁄

Table Question 6: Community

Deviation from reference party

Reference party

	AfD	CDU	SPD	FDP	Die Linke	Die Grüne
AfD	⁄	0.466	0.031*	0.217	0.886	0.615
CDU	0.466	⁄	0.152	0.626	0.544	0.769
SPD	0.031*	0.152	⁄	0.319	0.038*	0.065
FDP	0.217	0.626	0.319	⁄	0.247	0.398
Die Linke	0.886	0.544	0.038*	0.247	⁄	0.711
Die Grüne	0.615	0.769	0.065	0.398	0.711	⁄

Table Question 7: Party Members

Deviation from reference party

Reference party

	AfD	CDU	SPD	FDP	Die Linke	Die Grüne
AfD	⁄	0.034*	0.012*	0.124	0.255	0.184
CDU	0.034*	⁄	0.837	0.496	0.252	0.347
SPD	0.012*	0,837	⁄	0.346	0.135	0.215
FDP	0.124	0.496	0.346	⁄	0.616	0.792
Die Linke	0.255	0.252	0.135	0.616	⁄	0.811
Die Grüne	0.184	0.347	0.215	0.792	0.811	⁄

Table Question 8: Political View

Deviation from reference party

Reference party

	AfD	CDU	SPD	FDP	Die Linke	Die Grüne
AfD	∕	0.061	0.026*	0.353	0.717	0.184
CDU	0.061	∕	0.766	0.308	0.096	0.527
SPD	0.026*	0.766	∕	0.177	0.041*	0.338
FDP	0.353	0.308	0.177	∕	0.522	0.678
Die Linke	0.717	0.096	0.041*	0.522	∕	0.284
Die Grüne	0.184	0.527	0.338	0.678	0.284	∕
