# Supplementary material for: Political party affiliation, social identity cues, and attitudes about protective mask-wearing during the COVID-19 pandemic in Germany
Source: PLoS One. 2024 Jun 6;19(6):e0302399. doi: 10.1371/journal.pone.0302399 (PMC11156322; doi:10.1371/journal.pone.0302399)
Supplement: S1 Text — (DOCX) [file pone.0302399.s008.docx]

**Survey in English**

**General information:** With the help of this online survey, we would like to find out how best to implement public policies to contain the Covid 19 virus and similar viruses in the future. Specifically, we are interested in possible links between your response to the pandemic and your relationship with different political and social groups. To this end, we would like to hear from you in as much detail as possible about your experience of the pandemic, particularly in the period before vaccination became widely available (circa March 2020 to December 2020). The first three questions are designed to determine whether you are a suitable participant.

The estimated time for answering the questions is about 10-15 minutes.

Questions or other comments can be addressed to Dr. K. Magnus at ka.magnus@uke.de. If desired, these notes may be saved/printed. (Link here)

1. How old are you? ______ years

2. Which party would you probably vote for if the election were held next Sunday?

___ CDU/CSU

___ SPD

___ FDP

___ Left Party

___ Green Party

___ AfD

___ I won’t be voting

___ I don’t have the right to vote

___ No response/None of the above

3. Are you prepared to answer two open questions (approx. 3 to 4 sentences per answer)?

Yes ___ No ___

**Open-ended Questions**

1. How would you describe your experience of wearing a mask during the pandemic? Did wearing a mask trigger any particular feelings for you? (For example, were you upset or embarrassed? Did wearing a mask make you feel safer? Did you feel solidarity with others who were also wearing masks?) Please explain your experiences, thoughts, and feelings about this in as much detail as possible.

2. If a new virus or a new variant of Covid-19 for which there is no vaccine yet appeared, would you be willing to wear a protective mask in principle? Or would you wear a mask only under certain conditions (e.g. only in public places; only if hospitals were crowded; only if it was the law; only if you belonged to a risk group, etc.)? Please explain in as much detail as possible.

3. How did you feel when others refused to wear masks or when they did not wear the masks properly? Did you feel threatened? Did you sympathize with them? Or did you not care? Please explain in as much detail as possible.’

**Multiple-Choice Questions**

1. Did your decision to wear - or not to wear - a protective mask generally agree with the advice/behavior of the following people or groups?
Please mark one answer in each category (a-h).

a. with the advice of your doctor
___ don't know
___ not so much
___ sometimes
___ always/almost always
___ not relevant

b. with the recommendations of public health institutions (including institutes like the Robert Koch Institute)
___ don't know
___ not so much
___ sometimes
___ always/almost always
___ not relevant

c. with the behavior of your friends and acquaintances
___ don't know
___ not so much
___ sometimes
___ always/almost always
___ not relevant

d. with the behavior of your family members and relatives
___ don't know
___ not so much
___ sometimes
___ always/almost always
___ not relevant

e. with the behavior of your colleagues at work, university or school
___ don't know
___ not so much
___ sometimes
___ always/almost always
___ not relevant

f. with the behavior of members of social groups (e.g. church, sports club, etc.)
___ don't know
___ not so much
___ sometimes
___ always/almost always
___ not relevant

g. with the behavior of members of your political party
___ don't know
___ not so much
___ sometimes
___ always/almost always
___ not relevant

h. with the behavior of others who share your political views
___ don't know
___ not so much
___ sometimes
___ always/almost always
___ not relevant

2. If you wore a protective mask, what was your motivation?

Please mark one answer in each category (a-d).

a. to protect myself
___ not so relevant
___ somewhat relevant
___ particularly relevant

b. to protect others
___ not so relevant
___ somewhat relevant
___ particularly relevant

c. fear of punishment
___ not so relevant
___ somewhat relevant
___ especially relevant

d. social pressure
___ not so relevant
___ somewhat relevant
___ particularly relevant

3. Have you cancelled social contacts/ gatherings because there was a mask requirement?

___ no
___ rarely
___ sometimes
___ often

4. Are you a member of a political party? Yes _______ No _______

If yes, in which one?

___ CDU/CSU

___ SPD

___ FDP

___ Left Party

___ Green Party

___ AfD

___ Other

5. Which gender do you feel you belong to?

male ____ female ____ diverse ____

6. What is your monthly net household income, i.e. the total disposable income of all members living in the household after deduction of taxes and social security? (This includes social security and the like).

___ Less than 2,500 €
___ 2.500€ - 4.999 €
___ 5.000€ and more
___ Not specified/don't want to say
